# Challenges with the pursuit of ISO 15189 accreditation in a public health laboratory in Ghana

**DOI:** 10.4102/ajlm.v11i1.1448

**Published:** 2022-07-19

**Authors:** Seth Attoh, Francis K.M. Tetteh, Mary McAddy, Kingsley Ackah, Richmond Kyei, Marcus Moroti, Cynthia Boateng, Laurinda Adusu-Donkor, Joseph Boafo, Alhassan Yakubu, Sarah Kwao, Emmanuel Sarkodie, Nana-Banyin Koranteng, Monica A. Addo, Frederick Hobenu, Kwasi Agyeman-Bediako, Raymond D. Fatchu

**Affiliations:** 1 JM Wadhwani Department of Anatomical Pathology, Pathology Division Laboratory, 37 Military Hospital, Accra, Ghana; 2 Department of Microbiology, Pathology Division Laboratory, 37 Military Hospital, Accra, Ghana; 3 Department of Chemical Pathology, Pathology Division Laboratory, 37 Military Hospital, Accra, Ghana; 4 Department of Haematology, Pathology Division Laboratory, 37 Military Hospital, Accra, Ghana; 5 Department of Quality Assurance, Pathology Division Laboratory, 37 Military Hospital, Accra, Ghana

**Keywords:** ISO 15189:2012, accreditation, Strengthening Laboratory Management Towards Accreditation, laboratory, challenges

## Abstract

**Background:**

Accreditation is important for all medical laboratories, particularly public health laboratories in developing countries. Several laboratories in Ghana implemented the requirements of the International Organization for Standardization (ISO) 15189 but were unable to proceed to accreditation. This article describes the challenges faced by the Pathology Division Laboratory of the 37 Military Hospital, Accra, Ghana, during the acquisition of ISO 15189 accreditation and suggests solutions for a better approach.

**Intervention:**

Following ISO 15189 accreditation in 2017, an online survey was conducted between 01 and 30 March 2020 among the laboratory staff. Respondents were required to grade, on a scale of 0 (least) to 5 (most), the extent to which 16 key challenges influenced the process of obtaining accreditation. Key informant interviews were also held with laboratory personnel who were directly involved in the establishment of the quality management system in the laboratory and the accreditation acquisition process.

**Lessons learnt:**

Documentation, laboratory safety measures, laboratory management support, and reagent unavailability were estimated as the challenges that most affected the acquisition of laboratory accreditation. Challenges such as poor communication, staff apathy and workload had the least effect on the accreditation process. There was no difference in challenges identified between persons who worked in the laboratory before or after accreditation (*p* = 0.11).

**Recommendations:**

To surmount the anticipated challenges, there is the need for national strategic direction for laboratory accreditation, hospital and laboratory management support for the accreditation acquisition and maintenance processes, and sufficient technical assistance in the form of training and mentorship.

## Background

The quest to meet international standards, gain international recognition and acceptability, win the trust and confidence of clinicians and patients, improve quality of care, and improve the capacity to measure performance and streamline operations is driving medical facilities to pursue international accreditation.^1^ Accreditation and the pursuance of quality health systems in low- and middle-income countries have become critical in achieving universal health coverage.^2^

A well-structured and well-implemented healthcare system can result in better health outcomes and reduce health inequalities, hospitalisation rates, and hospitalisation costs.^3,4^ According to the World Health Organization, despite the huge investments made in the health sector globally, including the medical laboratory, health systems and laboratory practices still face significant challenges.^5^ In sub-Saharan Africa, several initiatives focused on medical laboratory improvement have resulted in few practical and sustainable outcomes.^6,7^ Key factors that negatively influence the accreditation process include the lack of prioritisation of accreditation, inadequate allocation of resources for attaining and maintaining accreditation, poor understanding of the importance of accreditation by both laboratory personnel and health authorities, and the high cost of the process.^8^

Between the years 2010 and 2013, Ghana enrolled 15 public sector medical laboratories in the Strengthening Laboratory Management Towards Accreditation programme to develop laboratory systems towards accreditation readiness and subsequent accreditation.^9^ At the close of the programme, three laboratories were awarded a four-star rating according to the Strengthening Laboratory Management Towards Accreditation grading system.^9^ However, despite the quality improvement within the respective laboratories, none has proceeded to accreditation due to major gaps threatening the acquisition of accreditation. Even for laboratories that have attained accreditation in other countries, weak hospital management support, inadequate documentation, inefficient equipment, irregular supply of reagents, little mentorship, and high staff turnover have been identified as bottlenecks to attaining and maintaining accreditation.^10,11^ As several African countries within the sub-region, including Ghana, pursue healthcare reforms to achieve universal health coverage, the need for national accreditation policies and systems has become a priority for several ministries of health.^12^

The Pathology Division Laboratory of the 37 Military Hospital became the first public sector laboratory to be accredited for methods in chemical pathology and haematology in Accra, Ghana, in 2017.^8^ Learning from its experiences and challenges, the laboratory has maintained its accreditation status over the years while gradually expanding its scope to include other departments in the division. The Microbiology Department also subsequently obtained International Organization for Standardization (ISO) 15189:2012 accreditation in 2019 from the Southern African Development Community Accreditation Service.

This article describes the challenges faced by the Pathology Division Laboratory of the 37 Military Hospital in Accra, Ghana, in the pursuit of ISO 15189:2012 accreditation and suggests solutions to the identified challenges.

## Description of the intervention

### Ethical considerations

Ethical clearance, with reference number 37MH-IRB/DS/IPN/417/2020, was received from the Institutional Review Board of the 37 Military Hospital, Accra, Ghana. Participants were required to respond to a consent section before voluntarily completing the online questionnaire. To protect the privacy of participants, no personal information was collected.

### Data collection

The study was conducted in the Pathology Division Laboratory of the 37 Military Hospital in Accra, the capital of Ghana, West Africa, which received ISO 15189 accreditation for haematology, chemical pathology, and microbiology test methods between 2017 and 2019. The laboratory also operates other departments, including histopathology, serology, and a blood transfusion service.

Key informant (KI) interviews were conducted with personnel who were directly involved in the establishment of the Quality Management System (QMS) of the laboratory and the accreditation acquisition process. These KIs included the officer-in-charge of the division, laboratory manager, quality manager, and heads of department for the haematology, chemical pathology, and microbiology departments. The KIs were coded (KI-1, KI-2, etc.) to maintain confidentiality. Interviews were digitally voice recorded after securing interviewee consent. Interviews were conducted in English, transcribed verbatim immediately thereafter, and then reviewed by members of the research team to ensure their validity. The main themes explored covered personnel, documentation, and management.

An electronic questionnaire was sent to all laboratory staff between 01 and 30 March 2020 to obtain staff opinions on the challenges with the laboratory’s accreditation acquisition process. The key challenges were selected based on other studies^10^ and the laboratory’s observed experiences during the ISO 15189 implementation phase. These covered challenges related to documentation, laboratory management support, laboratory safety, reagent availability, equipment availability, mentorship, support from doctors, hospital management support, staff attrition, staff qualification, staff strength, laboratory information management system, support from nurses, workload, staff apathy, and communication.

Participation in the study was voluntary. Participants were required to grade, on a scale of 0 (least challenging) to 5 (most challenging), the extent to which 16 key challenges impacted the accreditation acquisition process of the Pathology Division Laboratory. The three most challenging and three least challenging aspects of the accreditation process were identified. The maximum score attributable to each challenge is 520 (total number of respondents [*n* = 104] multiplied by the maximum score applicable [*n* = 5]).

### Data analysis

Data collected were processed and analysed in Microsoft Excel 2016 (Microsoft Corporation, Redmond, Washington, United States) and Stata IC/16 (StataCorp, College Station, Texas, United States). Summary descriptive statistics were used to describe the characteristics of the data set obtained from survey respondents. The number of responses on each challenge to accreditation was also represented as percentages for each score category. A multivariate regression model was used to compare the scores of key challenges between personnel who joined the Pathology Division Laboratory before accreditation versus after accreditation, adjusting for employee type, education, gender, and awareness of the accreditation status of the pathology division. *P*-values less than 0.0025 were considered statistically significant.

## Lessons learnt

A total of 106 of the 110 personnel working in the laboratory completed the structured questionnaire, representing a 96.4% response rate. There were two non-responses on time of joining the laboratory (before or after accreditation) and five non-responses on job title. More respondents (67/104, 64.4%) were present throughout the laboratory’s accreditation acquisition process compared to respondents (37/104, 35.6%) who joined the laboratory after the accreditation in 2017 (Table 1).

**TABLE 1:** Characteristics of study respondents (*N* = 106) at the Pathology Division Laboratory, 37 Military Hospital, Accra, Ghana, November 2020.

Characteristics	Number of respondents	Percentage (%)
**Time of joining the laboratory (*n* = 104)†**		
Before accreditation§	67	64.4
After accreditation¶	37	35.6
**Awareness of the laboratory’s accreditation status**		
Yes	104	98.1
No	2	1.9
**Sex**		
Male	80	75.5
Female	26	24.5
**Status**		
Military	88	83.0
Civilian	18	17.0
**Job title (*n* = 101) ‡**		
Medical laboratory assistant	9	8.9
Medical laboratory scientist	69	68.3
Medical laboratory technician	18	17.8
Haematologist	1	1.0
Histopathologist	3	3.0
Phlebotomist	1	1.0

† , Two participants did not respond;

‡ , Five participants did not respond;

§ , Staff working in the laboratory before and during the accreditation acquisition period in 2017;

¶ , Staff who joined the laboratory after the laboratory accreditation acquisition period in 2017.

**FIGURE 1:**
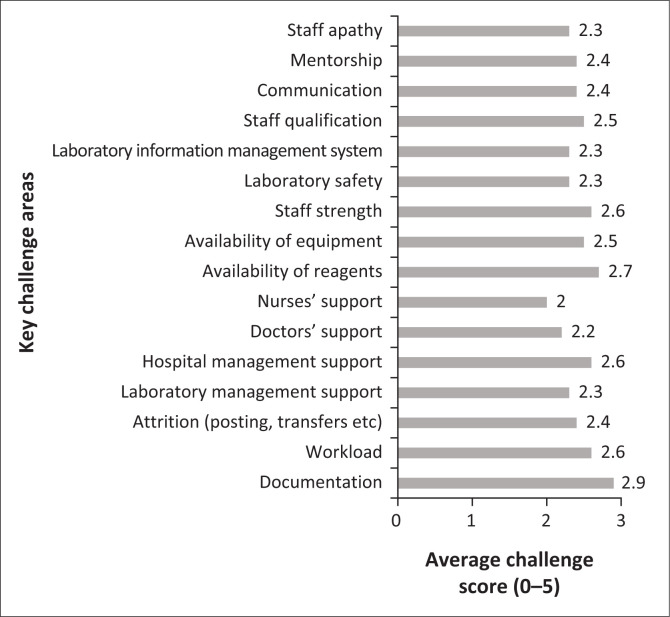
Scoring of key challenges to accreditation by laboratory staff at the Pathology Division Laboratory, 37 Military Hospital, Accra, Ghana, November 2020.

The pathway to ISO 15189 accreditation is marked with several challenges, especially in resource-limited environments. This, however, does not make it an improbable cause. Notable among the challenges to the accreditation process were laboratory personnel attrition, personnel attitude to change, service interruptions and logistic constraints, documentation, continuous external assessment outcomes, and size and complexity of the laboratory (Figure 1).

Documentation, availability of reagents, workload, and staff strength were among the highest-rated challenges. These challenges are consistent across different laboratories.^10^ Challenges such as nurses’ support, doctors’ support and staff qualification were rated among the least challenging.

The role of laboratory management in ensuring that the requirements of the international standards are understood by laboratory personnel is important to ensure collective buy-in and conformance. Where such inadequacies exist, mentorship would be needed to complement management roles in the application of the relevant standards.^8^

**FIGURE 2:**
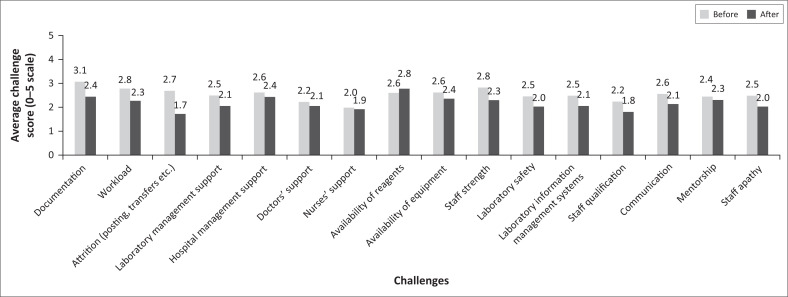
Comparison of the scores of key challenges to accreditation between personnel who joined the Pathology Division Laboratory, 37 Military Hospital, Accra, Ghana, before versus after accreditation, November 2020.

When controlled for employee type, education, gender, and awareness of the accreditation status of the pathology division, there was no statistically significant difference in responses between respondents who joined the laboratory before or after accreditation (Table 2, Figure 2). This is a clear indication that best practices implemented in the build-up to any accreditation process do not significantly change the laboratory’s processes, especially in laboratories where QMS may be seen as a burden. This ease of transition may be associated with the conscious effort to involve personnel in the accreditation process, thus increasing their level of satisfaction and making them more committed to the process.^13^ When staff are committed and involved in the accreditation process from the beginning, good commitment is more likely to persist throughout the life cycle of the laboratory.

**TABLE 2:** Multivariate regression model comparing the scores of key challenges between personnel who joined the Pathology Division Laboratory, 37 Military Hospital, Accra, Ghana, before versus after accreditation, November 2020.

Outcomes (challenges)	Unadjusted model			Adjusted model		
	Uβ	95% CI	*p*	aβ	95% CI	*p*
Documentation	−0.50	−1.15, 0.15	0.130	−0.61	−1.25, 0.03	0.063
Laboratory management support	−0.53	−1.17, 0.11	0.105	−0.54	−1.20, 0.12	0.109
Laboratory safety	−0.90	−1.54, −0.27	0.006	−1.01	−1.67, −0.34	0.003
Reagent availability	−0.31	−0.99, 0.37	0.366	−0.34	−1.06, 0.38	0.348
Equipment availability	−0.01	−0.61, 0.59	0.977	−0.07	−0.70, 0.56	0.833
Mentorship	−0.14	−0.75, 0.47	0.651	−0.30	−0.93, 0.32	0.338
Support from doctors	0.00	−0.59, 0.59	0.999	−0.18	−0.78, 0.43	0.560
Hospital management support	0.26	−0.37, 0.90	0.410	0.13	−0.53, 0.79	0.691
Staff attrition	−0.24	−0.89, 0.42	0.478	−0.29	−0.99, 0.41	0.409
Staff qualification	−0.45	−1.04, 0.13	0.130	−0.48	−1.10, 0.13	0.123
Staff strength (number of staff)	−0.28	−0.89, 0.33	0.365	−0.44	−1.07, 0.19	0.170
Laboratory Information Management System	−0.34	−1.01, 0.33	0.314	−0.38	−1.08, 0.31	0.278
Support from nurses	−0.34	−1.02, 0.35	0.334	−0.36	−1.08, 0.35	0.311
Workload	−0.31	−0.90, 0.28	0.296	−0.47	−1.07, 0.13	0.123
Staff apathy	−0.07	−0.71, 0.58	0.838	−0.15	−0.82, 0.52	0.654
Communication	−0.37	−1.02, 0.28	0.260	−0.49	−1.16, 0.17	0.145

Uβ, Unadjusted coefficients/difference in challenge scores (After – Before); aβ, Adjusted coefficients/difference in challenge scores (After – Before) controlling for employee type, education, gender, and awareness of the accreditation status of the pathology division; CI, confidence interval.

### Key informant interviews

#### Staff attrition

Critical among the challenges to the accreditation process was laboratory personnel attrition. KI-1 acknowledged:

‘As a military facility, military personnel are moved every now and then on military and allied duties resulting in significant reduction in staff numbers and consequent increase in workload on the remaining personnel.’ (K1-1, male, laboratory scientist)

As a result, persons trained and deemed competent in the quality system were lost and new persons introduced. This can be described as a ‘brain drain’ of laboratory personnel. According to KI-2 (female, laboratory manager), ‘between 2014 and 2017, five out of eight members of the laboratory quality steering committee members had been changed’. This certainly increased the amount of work the few remaining personnel had to do to support the quality system. To address this situation, the laboratory developed and deeply integrated a staff orientation and training programme to consistently update old and new staff with the status quo and requirements of the laboratory QMSs. Additionally, for every key appointment (e.g. quality manager, safety officer) there were two assigned deputies. Although these deputies had technical roles, they also understudied the key officer in preparation for taking over when one was moved. The attrition of personnel may constitute a significant form of brain drain in a laboratory that has built the capacity of its personnel to implement the requirements of the QMS. The effect of attrition is felt when the quality system is established around personalities and not as a functional system. Establishing a functional system is to ensure that developed policies and procedures are followed as required. However, there is the need to maintain a good level of stability of key personnel such as the quality manager and the quality team players to maximise the human resource capacity.

#### Personnel attitude to changing work routines

The attitude of personnel to changing work routines was another challenge that was apparent during the application process. According to KI-4:

‘Laboratory personnel considered the accreditation process and its requirement as additional work burden which added very little immediate financial benefits to one’s pocket hence the lack of enthusiasm towards the process.’ (K1-4, female, laboratory scientist supervisor)

We opine that the general perception among personnel is that the implementation of a quality system towards accreditation is entirely different from the ‘regular’ laboratory work. Hence, staff are more likely to go about their routine duties and only implement quality system requirements if they remember to do so. Both KI-3 and KI-4 emphasised:

‘The same personnel who are deployed on technical bench duties and processing of samples were the very staff involved in the numerous administrative and documentation tasks. They are simply overwhelmed with tasks and had the right to complain.’ (K1-3 & K1-4, laboratory scientist supervisors)

The routine duties of laboratory staff and the quality requirements associated with ISO accreditation are more interconnected than mutually exclusive as perceived. The perception of the two being separate stems from the situation where too much emphasis is placed on getting documentation done to the detriment of patient care or vice versa. Additionally, lack of motivation coupled with the extra duties increases that perception gap. Personnel are originally employed by their managers to run patient samples and churn out results. Therefore, any additional duty is expected to come with some extra, usually financial, remuneration. Where this fails to occur, as is the case in most public sector laboratories because they do not manage their own budget, enforcing such a change is met with firm resistance or indifference. This situation is however contrary to a study done in Lebanon where accreditation was found to be an impetus for better performance.^2^

It took several one-on-one mentorship and coaching sessions and in-house continuous education on ISO 15189 accreditation, the improvement process, the requirements of the Stepwise Laboratory Improvement Process Towards Accreditation checklist, good clinical laboratory practices, and laboratory QMS to get a lot more personnel on board with the process. According to KI-2:

‘The concerns of laboratory personnel regardless of how often or how much they complain is good feedback. Though we knew that indeed they were overwhelmed, everyone was overwhelmed. However, we initiated a staff of the month award scheme to motivate personnel.’ (KI-2, female, laboratory manager)

This ‘staff of the month’ award included a lunch package for the winner and a crate of drinks for the entire department, as well as the display of the winner’s photograph at the front desk.

#### Service interruptions and logistic constraints

Interruptions in the continuity of laboratory service was considered one of the challenges to the accreditation process. KI-1 explained:

‘Service interruptions usually occurred as a result of delays by suppliers to deliver reagents and other consumables, as well as bureaucratic procurement processes. These mostly resulted in reagent stock-outs. Most of the reagents and consumables required for testing are imported and are very capital intensive. Due to the amount involved and the foreign exchange transaction policies of the government of Ghana, it became even more difficult to carry out such procurement.’ (K1-1, male, laboratory scientist)

KI-1 acknowledged as a solution:

‘To ensure a more regular and continuous supply of reagents and consumables, some level of financial autonomy was given to the laboratory. An accountable imprest was also set aside for the laboratory. This brought to the fore the level of support offered by the hospital management.’ (K1-1, male, laboratory scientist)

The level of support and trust provided sufficient drive for the staff and management of the laboratory to improve their interest in the whole process and secure the coveted accreditation.

All KIs agreed that the absence of a dedicated budget for procurement of laboratory logistics was the main reason for the service interruptions occurring in the laboratory.

#### Continuous external assessment outcomes

In preparation for accreditation, self-checks and external assessments are needed to ascertain the readiness of the laboratory for the final accreditation assessment. According to KI-1:

‘We were subjected to several external assessments; however, it was as if the outcomes did us ‘more harm than good’. Despite the efforts put into preparing for external assessments, the scores were low and staff were demoralised. Different assessors almost reported different findings for the same requirement.’ (K1-1, male, laboratory scientist)

All KIs agreed that assessments (internal and external) are critical as required by the standard but that it is important to focus on managing the quality system rather than on the numbers or scores of an assessment.

#### Documentation

Constant documentation of activities is one of the key requirements of accreditations in general. The ISO 15189 standard requires the generation of evidence of the implementation of laboratory policies, processes, and procedures. KI-3 said:

‘It is like you have to document everything you do or do not do, this is hard, this is not something we are or were used to, hence the massive resistance.’ (K1-3, laboratory scientist supervisor)

In a time of evidence-based laboratory medicine, it is only imperative to document all activities as proof that they were carried out as required. The key role of conformance to documentation in any quality system cannot be overemphasised. KI-1 commented:

‘It is not possible for any laboratory to implement a quality system when the personnel who are the direct implementers of the system are not committed to the process. No matter how good the policies and processes are, if the people do not conform to it, it is useless.’ (K1-1, male, laboratory scientist)

This represented one of the biggest challenges to manage in an environment where documentation and conformance are not part of the culture. Group training and consistent one-on-one coaching with staff on the importance and benefits of documentation were conducted. Most importantly, results of documentation (e.g. occurrence, quality indicators, temperature monitoring, etc.) were periodically reviewed and communicated to staff to help them appreciate the need for and benefits of keeping quality records.

#### Size and complexity of the laboratory

KI-5 (laboratory scientist) stated that ‘the medical laboratory, to the ordinary person, may be perceived as a small space within which simple tests are performed’, and further opined that the converse rather is true, stating that ‘the laboratory is a large complex network of several units and departments involved in an interconnected series of test activities’. This was a major challenge. The laboratory has five testing departments, collectively performing over 100 test panels and generating results for over 300 analytes. ‘At a point we were confused, not knowing exactly what was going on because they were just too many’, KI-2 (laboratory manager) said. The initial attempt to get all methods accredited at once was not working. The burden of work was overwhelming due to the size and complexity of the laboratory. Therefore, the laboratory adopted a stepwise approach to attaining its much-desired accreditation. This phased approach implied prioritising the methods to be accredited and working at accreditation in smaller chunks. Chemical pathology and haematology were therefore chosen for the initial accreditation process, with the subsequent addition of microbiology a year later.

#### No historical precedence

All KIs acknowledged that the accreditation of a public sector laboratory in Ghana was considered highly improbable. This made it even more difficult for laboratory staff to be convinced that it was possible to be accredited as a public sector laboratory. As explained by KI-2:

‘Staff always made reference to other bigger hospitals in Ghana and in the West African sub-region that if those facilities were not accredited though better placed to be, how was it going to be possible for the military hospital which is resource-stricken be accredited.’ (K1-2, female, laboratory manager)

However, it was clear that although there was no historical precedence, with the needed guidance and mentorship, it was possible to meet the requirements for accreditation.

According to KI-1 and KI-5:

‘Effective mentorship is a pivotal element for any public sector laboratory seeking accreditation. In the absence of a historical precedence, our mentorship program guided the development of policies and process and further assisted in the implementation, conformance and the review process.’ (K1-1 & K1-5, laboratory scientists)

In a country where the motivation of the government towards laboratory accreditation is weak, securing accreditation for any public sector laboratory is usually a huge challenge.^14^ The inadequacies in the national support for accreditation result in the inadequate dedication of resources (human, capital and infrastructural) towards attaining and maintaining accreditation. While a national focus is required, hospital management support, including support from other healthcare workers such as doctors and nurses, cannot be overemphasised.^8^ Management support plays a key role in the accreditation process in resource-limited settings, and the engagement of hospital management with laboratory management accelerates the accreditation acquisition process.

Perspectives about the challenges with securing accreditation vary between laboratory personnel and management staff. While laboratory personnel identified excessive documentation, weak laboratory management support, and inadequate safety as the main challenges to accreditation, management personnel identified staff attrition, personnel attitudes, and the size and complexity of the laboratory facility as the main challenges. However, both groups of personnel identified excessive documentation and service interruption as key challenges. There is a need for constant feedback across levels of hierarchy to get everyone on the same page in the quest for accreditation. That way, the resources, although limited, can be better apportioned to address common challenges.

### Limitations

The study was limited to staff of the Pathology Division Laboratory so the perspectives of hospital management, other hospital staff and clients were not solicited. The KI interviews did not include personnel whose testing activities were not within the scope of accreditation. Some bias may therefore be inherent in the responses.

## Recommendations

To make laboratory accreditation more relevant and, consequently, surmount the anticipated challenges, there is the need for national strategic direction for laboratory accreditation, hospital and laboratory management support for the accreditation acquisition and maintenance processes, and sufficient technical assistance in the form of training and mentorship.
